# Development, Characterization, and Evaluation of Novel Broad-Spectrum Antimicrobial Topical Formulations from* Cymbopogon martini* (Roxb.) W. Watson Essential Oil

**DOI:** 10.1155/2018/9812093

**Published:** 2018-09-10

**Authors:** Negero Gemeda, Ashenif Tadele, Hirut Lemma, Biruktawit Girma, Getachew Addis, Birhanu Tesfaye, Abiy Abebe, Worku Gemechu, Kidist Yirsaw, Frehiwot Teka, Christina Haile, Aliyi Amano, Samuel Woldkidan, Bekesho Geleta, Asfaw Debella

**Affiliations:** Traditional and Modern Medicine Research Directorate, Ethiopian Public Health Institute, P.O. Box 1242, Addis Ababa, Ethiopia

## Abstract

**Background:**

Skin infections were the most frequently encountered of all infections and the 4th leading cause of nonfatal disease burden. Topical drugs have been used for the management of skin infections. The growing concern of drug resistance to the topical agents has warned the need for continuous development of novel drug. Essential oils are the best candidate for new drug with different mode of action and target as they are rich in chemical constituents.

**Objective:**

To evaluate and develop safe and effective topical antimicrobial formulations from essential oil of* Cymbopogon martini. Method.* Essential oil was extracted using hydrodistillation aerial part* C. martini *and topical formulations were prepared in five different semisolid bases.* In vitro* antimicrobial investigations were performed on essential oil and topical formulations. Skin sensitizations of the formulations were evaluated using guinea pig maximization.

**Results:**

The essential oil of* C. martini *has shown broad-spectrum antimicrobial potency against all tested organisms with MIC value ranging from 0.65 to 10 *μ*g/ml. Absolute inhibitions of growth of fungi were observed against* Trichophyton mentagrophytes *and* Trichophyton rubrum* at concentrations above 1% of oil and against* Microsporum canis *and* Trichophyton verrucosum* at a concentration of 4% oil. Among topical formulations, the highest antimicrobial activity was recorded in hydrophilic ointment followed by macrogol blend ointment. The antimicrobial activity of oil was higher in fungal pathogen compared to bacteria. Gram positive bacteria were more sensitive than gram negative bacteria. Hydrophilic and macrogol blend ointment containing 5% oil did not produce any skin sensitization on guinea pigs.

**Conclusion:**

In conclusion, topical formulations of* C. martini* essential oil can be alternative topical agents with safe broad-spectrum activity for the treatment of skin disorder. Further studies should focus on shelf life study and clinical study of the product.

## 1. Introduction

Naturally, skin provide mechanical barrier to the body against the invasion by microorganisms through protective keratinous surface, which allows the removal of microorganisms via sloughing off keratinocytes and acidic sebaceous secretions [1]. However, break in the skin in case of surgery, cuts, puncture, decubitus ulcer, animal or insect bites, thorn and needle pricks, or burns can result in skin infections [2]. The spectrum of infection ranges from asymptomatic colonization to bacteraemia, fungemia, and death [3]. Common skin infections caused by microorganisms include carbuncles, furuncles, cellulitis, impetigo, boils, folliculitis, ringworm, acne, and foot odor [4].

Skin disease is one of the most frequently encountered infections for which people commonly seek medical intervention, particularly in developing countries [5]. The magnitude of the infection is as high up to 76% and can occur in all individuals with higher rate of infection in immune-compromised individuals [6]. Skin infections were the 4th leading cause of nonfatal disease burden when comparing absolute DALYs/YLDs [6]. In 2016, fungal skin diseases were among the top four of the ten causes with the highest incidence by accounting for (2·10 billion, 1·88 billion to 2·34 billion) [7]. Its detrimental effects on health range from physical incapacity to death [8]. Children and their families often bear the brunt of this disease burden [5, 9]. Skin infections are prevalent in developing countries due to various factors including poor socioeconomic status, high population densities, and poor sanitary conditions [4–6, 9].

The management of skin infections requires treatment by oral and/topical antimicrobial formulations [10, 11]; however, there has been negative treatment outcomes due to abundant harmful effects associated with synthetic antimicrobials and due to the emergence of resistance in common skin pathogens such as* S. aureus *resulting as methicillin-resistant* Staphylococcus aureus *(MRSA),* Pseudomonas aeruginosa,* and other such strains [12]. Treatment has therefore become a challenge and is often not successful [12]. There was a report of infections that are unresponsive to all known antibiotics [13]. This threat has become so severe that simple ulcers now require treatment with systemic antibiotics [12] and simple cut on the finger could result in death by infection [12]. The World Health Organization (WHO) has warned that common infections may be left without a cure as we are headed for a future without antibiotics [14]. Therefore, one of the candidates available to use is the oldest form of medicine, natural product for the treatment of skin disorder [15, 16]. Essential oil (EO) with its complex chemical constituents can be the best source to develop novel topical antimicrobial formulation with different mode of action.

Ethiopia is rich in biodiversity and traditional medicine practices and the use of traditional medicine/natural product especially medicinal plants for the treatment of different aliment including skin disease has been very popular in the community [17]. A review of readily available ethnobotanical literature has reported over 229 plant species which belong to 67 families that are used in Ethiopian Traditional Medicine for the treatment of various dermatological disorders [18].* C. martini* was among the ethnobotanically reported aromatic plants and previous study has reported its antimicrobial activity of its EO. However, antimicrobial activity of topical formulations has not been evaluated against skin disease causing pathogens. Taking into account the availability and acceptability of the practices in the Ethiopian society [19], our study was aimed at evaluating and developing safe and effective antimicrobial agents from EO of* C. martini* for the management of various dermatological disorders caused by major skin disease causing microorganisms.

## 2. Materials and Methods

### 2.1. Materials

The chemicals, media, and solvents used in this study were anhydrous sodium sulfate, cetostearyl alcohol, hard paraffin, liquid paraffin, propylene glycol, stearyl alcohol, and wool fat and were purchased from Sigma-Aldrich (Germany). Polyethylene glycol (PEG) 4000 and PEG 600 were obtained from BDH (England). Sodium lauryl sulfate was purchased from LABORT Fine Chemicals (India) and white petrolatum USP was purchased from EPHARM (Ethiopia). Muller-Hinton agar, Muller-Hinton broth, nutrient agar, nutrient broth, Sabouraud dextrose agar, Sabouraud dextrose broth, tryptone soya, agar and tryptone soya broth were purchased from Oxoid (UK). Yeast extract was purchased from LABORT (India).

### 2.2. Methods

#### 2.2.1. Collection of Plant Materials and EO Extraction

*C. martini *(aerial part) was collected from the botanical garden of Traditional and Modern Medicine Directorate (TMMRD), Ethiopian Public Health Institute (EPHI), Addis Ababa, Ethiopia. The identity of the plant was authenticated by Getachew Addis (Ph.D.), lead researcher botanist in the directorate and voucher specimens (HTMMRD 001NGGA) of* C. martini* were deposited in the Herbarium of the TMMRD. Following collection and identification, fresh aerial parts of* C. martini *(250 g) were chopped and placed in a 5 L round-bottom distillation flask and the plant material was wetted with 3 L distilled water. The EO (EO) was obtained by hydrodistillation using Clevenger-type apparatus for continuous 3hours. The oil was taken from the upper layer. The excess aqueous layer was further portioned using dichloromethane to extract and enrich the EO from the water layer. The organic layer (dichloromethane extract) was filtered and dried with anhydrous sodium sulfate and concentrated using rotary evaporator to give the crude EO.

#### 2.2.2. Test Organisms

The following bacterial and fungal strains were used in the study:* Klebsiella pneumoniae* (ATCC 13883 and clinical isolate),* Pseudomonas aeruginosa *(ATCC 27853 and clinical isolate),* Staphylococcus aureus* (ATCC 25923 and clinical isolate),* Streptococcus pyogen* (ATCC 19615 and clinical isolate),* Aspergillus niger* (ATCC 10535 and clinical isolate),* Candida albicans* (clinical isolates),* Microsporum canis *(clinical isolate),* Trichophyton mentagrophytes *(ATCC 18748 and clinical isolate)*, Trichophyton rubrum* (ATCC28188), and* Trichophyton verrucosum *(clinical isolate). The clinical isolates of the test organisms were obtained from Bacteriology and Mycology Directorate, EPHI.

#### 2.2.3. Commercial Topical Antimicrobial Products

Gentamycin cream (1% gentamycin HOE Pharmaceuticals Sdn. Bhd, Batch No.: 55964015, Malaysia) and Fungigen® cream (2% miconazole BP cream, Batch No: CJ07003, Galentic Pharma India) were obtained from local drug retail outlets in Addis Ababa. The commercial topical drugs were used as positive control in the assay.

#### 2.2.4. Preparation of Topical Formulations

Five different formulation bases were prepared by the fusion method except for the white soft paraffin which was prepared in accordance with the formula given in Table 1. Different concentration (v/w) of the EO topical formulations was prepared by incorporating the oil into the soft mass of the different dermatological bases. The bases, without the EO, were used as negative controls in the assay.

#### 2.2.5. Physical Evaluation of the Topical Formulation

*(1) Organoleptic Examination.* The prepared formulations were inspected visually for their physical appearance, color, texture, phase separation, and homogeneity. Except for homogeneity and texture, all the characteristics were inspected visually; however, the homogeneity and texture were tested by pressing small quantity of the formulated product between thumb and index finger. The consistency of the formulation and presence of coarse particles were used to evaluate the texture and homogeneity of the formulation.

*(2) Creaming and Coalescence.* A 10 g sample of each formulation was placed in a beaker and stored at room temperature for 3 months. Their physical stability was determined after one week and one- and three-month storage.

*(3) Centrifugation.* A 10 g portion of each formulation was placed in a centrifuge tube (1 cm diameter) and centrifuged at 2000 rpm for 5, 15, 30, and 60 min. Then the phase separation and solid sedimentation of the samples were evaluated.

*(4) Thermal Cycle Test.* The portion was stored at 5°C for 48 h and then at 25°C for 48 h. The procedure was repeated 6 times and then their stability and appearance were evaluated.

*(5) Freezing and Thawing.* Twenty-gram portion of each formulation was stored periodically at 50°C and 4°C for 48 h each. The procedure was repeated six times and then the samples were checked regarding their appearance and stability.

*(6) Determination of pH. *A suspension of each portion in 1% potassium nitrate solution was prepared and its pH was determined. A magnetic stirrer was used to produce homogeneity.

*(7) Spreadability.* The spreadability was determined by applying weight to glass slides into which formulation placed, and time in seconds required to separate the slides was noted [20]. The spreadability was calculated by using the formula, S = M.L/T (1), where, S is spreadability, M is weight tide to upper slide, L is length of glass slide, and T is time taken to separate the slide completely from each other.

#### 2.2.6. Antimicrobial Assays

*(1) Minimum Inhibitory Concentrations (MIC).* The MIC value of* C. martini* EO was determined using agar dilution assay [21, 22]. Twofold serial dilutions of EO solution in Muller-Hinton agar (for bacteria) and Sabouraud dextrose agar (for fungi) have a final concentration of EO ranging from 0.25% (v/v) to 4% (v/v). The assays were performed in triplicate. Control plates, containing no EOs, were run simultaneously. The agar surface of the plates containing the dilution of EOs and the control plate were inoculated by standard inoculum (having optical density of 0.08 -01 at 625 nm wavelength) of overnight (bacteria and yeast) and five-day (mold and dermatophyte) grown cultures. The plates were incubated at 25°C for seven days (for fungi) and 37°C for 24 h (for bacteria). After incubation, the end-points for the EO were determined by placing plates on a dark background and observing the lowest concentration that inhibits visible growth, which is recorded as the MIC.

*(2) Agar Well Diffusion for Antimicrobial Activity of EO.* Agar well diffusion assay was performed to using standard technique [21–23]. (1) Agar well diffusion for EO: A volume of 0.2 ml suspension of overnight microbial having standard turbidity of the respective fungus and bacteria was added to a test tube containing 20 ml of molten and cooled (45°C) Muller-Hinton agar (for bacteria) and Sabouraud dextrose agar (for fungi). The agar suspension of organisms was thoroughly mixed by vortex mixer and poured onto the respective sterile Petri dish and allowed to solidify. After solidification, one hole with 10 mm diameter was made by cutting out a plug of agar from the center of each plate using sterile cork-borer. About 150 *μ*l of EO solutions was transferred to the prepared well. The plates were allowed to stand for 2 hours at room temperature in order to allow diffusion and incubated at 37°C for 24 h (for bacteria) and 25°C for 7 days (for fungi)). After incubation, the diameter of zones of inhibition was measured.

*(3) Antimicrobial Activity of Topical Formulations.* Release antimicrobial activities of topical agents were performed using the method adopted in our laboratory [23, 24]. Fifteen milliliters of molten and cooled molten and cooled (45°C) Muller-Hinton agar (for bacteria) and Sabouraud dextrose agar (for fungi) was added to sterile Petri dish and allowed to solidify. After solidification, one hole with 10 mm diameter was made by cutting out a plug of agar from the center of each plate using sterile cork-borer. About 0.1 ml (0.2 g) of designated topical formulations was added to each well. A volume of 0.2 ml suspension of overnight microbial having standard turbidity of the respective fungus and bacteria was added to a test tube containing 7 ml of respective molten agar. The agar suspensions of organisms were thoroughly mixed by vortex mixer and poured into a plate containing topical formulation to make an agar overlay. After the plate was solidified for 2-3 minutes, it was inverted and allowed to stand for 1 hour at room temperature and incubated at 25°C for 7 days (for fungi) and 37°C for 24 h (for bacteria). After incubation the diameter of zones of inhibition was measured.

#### 2.2.7. Effect of Temperature on the Activity of* C. martini* EO

The effect of temperature on antimicrobial activity of* C. martini *EO was tested according to Shahi and Shahi [25], using poisoned food technique at concentration of respective MIC of each tested fungal pathogen using Sabouraud dextrose agar as nutrient medium [21, 25]. Five lots of EO were kept in small brown vials, each containing 5 ml of EO; these were exposed to room temperature of 40, 60, 80, and 100°C in incubator for an hour. Five-day-old fungal culture was punched aseptically with a sterile cork-borer of generally 5 mm diameter. The fungal discs were then put on the gelled agar plate. The agar plates were prepared by impregnating desired concentration of EOs at their respective MIC for each organism at a temperature of 45°C. The plates are then incubated at temperature of 25°C for 7 days. Colony diameter was recorded by measuring the two-opposite circumference of the colony growth. The result was expressed in percentage inhibition of fungal growth by comparing the colony diameter of poisoned plate (EO) and nonpoisoned plate (with distilled water) and calculated using the formula given below:

(1) Percent Growth Inhibition:Growth in Control−Growth in TestGrowth in Control×100

#### 2.2.8. Skin Sensitization Test of the Formulated Products

Skin Sensitization test was conducted by the Guinea Pig Maximization Test (GPMT) according to Organization for Economic Cooperation and Development (OECD) guideline number 406 [26] by using the BUEHLER TEST method [27]. All animals were randomly grouped into treatment and control groups and acclimatized to laboratory conditions for a period of one week before the actual experiments. All animals had free access to standard diet and water and they were treated humanely throughout the study period according to International Guidelines of Laboratory Animal Care and Use [28]. Ten animals (5 male and 5 female) in each treatment and control groups were used. The test area was cleared of hair. A filter paper (2 × 4 cm) is fully loaded with 0.5g topical formulations and positive control and applied to the test area and held in contact by an occlusive dressing for 48 hours. In negative control groups only bases were applied. Observations of treated and control groups were made to record skin reaction according to the grades of Magnusson and Kligman Grading Scale for the Evaluation of Challenge Patch Test Reactions using ordinal scale of 0 = no visible change, 1 = discrete or patchy erythema, 2 = moderate and confluent erythema, and 3 = intense erythema and swelling is used [26].

#### 2.2.9. Statistical Analysis

All the measurements were replicated three times for each treatment and data were entered into excel spreadsheet and are presented as mean ± SD. One-way analysis of variance (ANOVA) followed by Tukey's* post hoc* test was used for statistical evaluation. P values less than 0.05 (p<0.05) were considered significant.

## 3. Results

### 3.1. *In Vitro* Antimicrobial Activity

#### 3.1.1. Antimicrobial Activity of EO

*(1) Minimum Inhibitory Concentrations.* Table 2 showed the minimum inhibitory concentration of the EO of* C. martini *against skin disease causing bacterial and fungal strains of standard and clinical isolates. The oil has superior biological activity against fungal pathogens compared to bacteria. In bacterial pathogens the EO of* C. martini *have showed potentially highest inhibitory activity against the growth of* Streptococcus pyogenes *with MIC of 2.5 *μ*g/ml.* Trichophyton *species were the most susceptible pathogens in fungal category, with the MIC value of 0.65 *μ*g/ml. Standard strains of all bacterial strains are susceptible to the challenge of EOs, as compared to clinical isolates except for* Staphylococcus aureus *where both clinical and standard stains are susceptible to the same MIC value of 5 *μ*g/ml. Higher MIC of oil was recorded against clinical isolates compared to the standard strains of* Pseudomonas aeruginosa *and* Klebsiella pneumonia*. Similarly, among the tested fungal pathogen, less susceptibility was recorded by both clinical and standard strains of* Aspergillus niger* with MIC value of 2.5*μ*g/ml.

*(2) Antimicrobial Activity Using Agar Well Diffusion Assay.* In agar well diffusion method to determine antibacterial activity of* C. martini *EO at five different concentrations against skin disease causing bacterial strains, the oil has shown broad-spectrum bacterial growth inhibitory activity against all skin disease causing bacterial pathogens at the tested concentrations (Table 3). Gram positive organisms have showed higher susceptibility to the exposure of all tested concentrations of the oil compared to the gram negative bacteria. The highest growth inhibitory activity of the oil was exhibited at 4% concentration against* S. pyogenes *clinical isolate, while the least was seen at 0.25% concentration against* P. aeruginosa *clinical strains.

The EO of* C. martini* displayed significantly high inhibitory activity against all tested skin diseases causing fungi with inhibition zone ranging from 10.5 ± 0.0 to greater than 90 mm (Table 4). Strong inhibition was recorded against all dermatophytes (*M. canis*,* T. mentagrophytes, T. rubrum, *and* T. verrucosum) *at all tested concentrations followed by standard strains of mold,* Aspergillus niger,* with the inhibition diameter of 70 mm at a concentration of 4%* C. martini* EO and yeast,* Candida albicans*. Absolute inhibitions of growth of fungi were recorded against* T. mentagrophytes *and* T. rubrum* at concentrations of 1%, 2%, and 4%* C. martini* EO and against clinical isolates of both* M. canis *and* T. verrucosum* at a concentration of 4% oil.

#### 3.1.2. Antimicrobial Activity of Topical Formulations

Table 5 shows antibacterial activity of five different topical ointments containing EOs of* C. martini *in three different concentrations. From all formulation hydrophilic ointment has the highest antibacterial activity at all formulated concentrations against all the test bacteria with inhibition zone diameter ranging from 4.0±1.0 to 33.3±0.5 (Table 5). Comparatively, both hydrophilic ointment and macrogol blend ointment showed significant antibacterial activity against all the tested skin disease causing bacteria. On the other hand, macrogol cream ointment, simple ointment, and white petrolatum ointment formulations of* C. martini* showed no antibacterial growth inhibition activity against the tested bacteria.

Antifungal activity of topical formulations containing* C. martini *EO at a concentration of 1%, 1.5%, and 2% in five different formulation bases was displayed in Table 6. The table represents that hydrophilic ointment and macrogol blend ointment had fungal growth inhibitory activity while the rest of the formulation (macrogol cream ointment, simple ointment, and white petrolatum ointment) have no inhibitory effects on the growth of tested skin disease causing fungi. Superior antifungal activity was recorded by hydrophilic ointment at all tested concentration and organisms with the inhibition zone diameter ranging from 21.0±1.7 to greater than 140 mm. Dermatophyte was the most susceptible organisms to the treatment of both hydrophilic ointment and macrogol blend ointment containing* C. martini *EO. Complete inhibitions of fungal growth were recorded against clinical and standard strains of* T. mentagrophytes* at all concentration except 1% against clinical isolates of the organism. Similarly, hydrophilic ointment and macrogol blend ointment exhibited inhibition of the entire plate against* T. rubrum *at a concentration of 1.5% and 2% and against* T. verrucosum* exposed to hydrophilic ointment containing 1.5% or 2% EOs and to macrogol blend ointment containing 2% EO.

### 3.2. Effect of Temperature on Activity of C.* martini* EO

Table 7 shows the effect of temperature on antifungal potency of* C. martini* EO exposed to different temperatures, namely, room temperature, 40, 60, 80, and 100°C for one hour in the incubator. The exposure of* C. martini* EO to all tested temperatures caused no change in the potency of the oil against all tested fungal strains at their particular MIC.

### 3.3. Skin Sensitization and Irritation

The skin sensitization and irritation score of topical formulations containing* C. martini* EO were shown in Table 8. The test for skin sensitization for 5% topical formulation of* C. martini* on hydrophilic and macrogol blend bases did not produce any skin sensitization and irritation on guinea pigs. Scale of no visible change was observed according to the Magnusson and Kligman Grading Scale in guinea pigs treated with 5% EO topical formulations compared to the standard sensitizer sodium lauryl sulfate 70% sensitization rate by producing intense erythema and swelling.

### 3.4. Physical Evaluation of Topical Formulations

Table 9 showed the physical characterization of the formulated products. The result showed that the formulations had good appearance, all the formulations are homogenous, and they all are stable to creaming and coalescence test and centrifugation test. Formulation of C.* martini* oil in hydrophilic ointment base, macrogol ointment base, and white petrolatum base has shown stability in thermal cycle test and in freezing and thawing test. No weight loss was recorded in all formulations. Hydrophilic ointment and macrogol blend ointment formulations showed good release with pH value of 5.8 and 6.2 and spreadability values of 14.7 and 10.5, respectively. In many of the test parameters both hydrophilic ointment and macrogol blend ointment have better characteristics.

## 4. Discussions

The use of aromatic plants preparations for the treatment of various ailments including skin disease was dated back to the time of antiquity [29]. Even in this era of medical advancement, their application as aromatherapy was quite popular [30]. This has increased the interest and demand to develop antimicrobial products from EO to treat skin infections that could substitute synthetic antimicrobial to which the organisms had developed resistance [30]. Hence, the scope of this study is to assess and evaluate antimicrobial potency of* C. martini* EO against skin disease causing pathogens and develop topical formulations EOs.

In this study significant potency of* C. martini* EO against skin disease causing bacterial and fungal pathogens was observed in agar dilution assay. A very low MIC value was recorded in almost all test organisms. The broad-spectrum antimicrobial activity of* C. martini* EOs would be attributed to different mode of action as the oil is known to have complex mixtures chemical compounds made of sesquiterpene and monoterpene hydrocarbons and alcohols, ketones, aldehydes (their oxygenated derivatives), and other chemical families like fatty acids, oxides, and sulphur derivatives [31]. Chemical composition, amount, structural configuration, and their functional groups and possible synergistic interactions between components would be responsible for this remarkable activity of* C. martini* EO [32, 33]. In addition, earlier study has reported antimicrobial potency of geraniol (terpene alcohol), a major constituent in this EO [34, 35].

The result also showed strong efficacy of the EO against tested fungi compared to bacteria and against gram positive bacteria than to gram negative bacteria with higher resistance to ward* P. aeruginosa *and* K. pneumoniae*. Even though many mechanism actions (inhibition of cell wall formation, inhibition of efflux pump, cell membrane disruption, and dysfunction of the fungal mitochondria) were involved in the biological activity of EOs, the superior potency of the* C. martini* EO in this study against fungal pathogen compared to bacteria pathogen may be due to inhibition of ergosterol synthesis [36]. Previous study has reported the major constituents of* C. martini *essential oil and geraniol and inhibits ergosterol biosynthesis, a major constituent in fungal plasma membrane [34]. Out of the test fungi, a very high MIC was observed against* Aspergillus niger*. Similar finding were reported from various previous studies [37, 38].

The findings from agar well diffusion techniques have shown that the EO of* C. martini* have strong antibacterial property at all tested concentrations in dose-dependent manner. Previous study has shown similar results, upon the increment of* C. martini* EO concentration and increased inhibition of the growth of both gram positive and gram negative bacteria, indicating dose-dependent response [39]. This strong potency of the oil can be attributed to the presence of large group of components, allowing the oil to have diversity of mechanism of action and targets [40]. Previous studies have linked the mechanism of action of EO to its lipophilic nature resulting in increased cell permeability and leakage of cell constituents which can result in different alteration to bacterial cell through cell wall and membrane disturbance, ATP losses, inhibition of protein synthesis, pH disturbance, intracytoplasmic changes, DNA damage, and inhibition of cell-cell communication (quorum sensing) [40].

Higher inhibitions were recorded against gram positive bacteria, particularly* S. pyogenes *at all tested concentrations compared to gram negatives especially* P. aeruginosa*. This may be attributed to the presence of additional outer membrane acting as an effective barrier for amphipathic agent [40] and overexpression of efflux pumps responsible for innate antimicrobial resistance [41]. Similar findings were reported by previous study indicating that gram positive bacteria are more susceptible to* C. martini* EO than gram negative bacteria [39, 40].

The* C. martini* EO had showed strong dose-dependent broad-spectrum antifungal activity against* C. albicans*,* A. niger,* and dermatophytes. Complete inhibitions were recorded at a concentration of 4% against all dermatophytes and at a concentration of above 1% against* T. mentagrophytes *and* T. rubrum*. The strong antifungal activity of* C. martini* to different group of fungi could be due to the constituents of EO, which may have exerted their efficacy towards the fungal pathogen individually or in a synergetic manner [32]. Moreover, these constituents would be responsible for multitarget action on fungal cell, which would have led to the permanent damage of the fungal cell [38, 40, 42]. In addition, several works have reported the antifungal, particularly antidermatophyte activity of geraniol, the major constituent of* C. martini* EO [34, 35].

In the antimicrobial activity of topical formulations prepared from EO of* C. martini*, hydrophilic ointment showed the highest potency followed by macrogol blend ointment against all tested bacteria and fungi at every tested concentration. In contrast, there was no activity in macrogol cream ointment, simple ointment, and white petrolatum ointment formulations. From this it is evident that the oil was easily diffused or the bioactive constituents in the formulations was easily released and inhibited the growth of the tested microorganism. Previous study has reported that the efficacy of formulation depends on the nature of bases, in which the release of bioactive compound from the base depends on its affinity for the base than media used for antimicrobial assay and the viscosity of the formulation base [24]. The results have showed significantly higher activity of* C. martini* EO formulation in hydrophilic ointment and macrogol blend ointment base. Various reports from previous study have reported superior efficacy in hydrophilic ointment formulation and macrogol blend ointment formulation of other EO [24]. Moreover, the increased activity of the Hydrophilic ointment formulation can be attributed to the presence of sodium lauryl sulfate, which is used as a surfactant, has biological activity against bacterial and fungal strains, and is also used pharmaceutically as a protective skin cleaner as well as in medicated shampoos [43], while the better antimicrobial activities of the macrogol blend ointment formulation may be due to the fact that polyethylene glycols have an excellent solubility in water and can enhance the diffusion bioactive constituents in to the media which are more hydrophilic [44]. On the other hand, the absence of efficacy of* C. martini *EO in the remaining three formulation bases, namely, macrogol cream ointment, simple ointment, and white petrolatum ointment base, can be attributed to the lipophilic affinity of the EO for paraffin, which impairs the release of its active constituents into the more hydrophilic agar medium. Moreover, paraffin is much more viscous than* C. martini *oil and this would further hinder diffusion of the active principle from the oil into agar medium.

Our result has shown significantly higher efficacy of topical formulations (hydrophilic ointment and macrogol blend ointment) containing* C. martini* EOs than the used commercial topical antimicrobial drugs (1% gentamicin cream for bacteria and 2% miconazole cream for fungi) at a concentration greater than 1% of preparation against almost all test organism used in the study. The efficacy can be attributed to the presence antimicrobial active major constituent geraniol and the presence of various bioactive constituents in the EO of* C. martini*, which would have imposed additive toxic effect towards the skin disease causing microorganism. Moreover, the small molecular weight of chemical constituents in the EO of* C. martini* EO may have resulted in easy diffusion of the molecule in agar, as light compounds diffuse larger than heavy compound. Various previous studies have reported that a bioactive principle with light molecular weight diffuses easily in agar medium than constituents with heavy molecular weight [45].

The biological activity of EO of* C. martini *was found stable to the exposure of high temperatures. This may be attributed to the thermodynamic stability of bioactive components present in the EO and also partly due to temperature used during hydrodistillation to bear the EO from the start. It has been shown in several reports that the antimicrobial activities of EO were stable in contrast to varying chemical composition to the exposure of different temperature [46].

The result of skin sensitization and irritation of 5% topical formulations containing* C. martini* EO showed no sign of any dermal sensitization and irritation on guinea pigs compared to 70% sensitization (intense erythema and swelling) in guinea pigs treated with sodium lauryl sulfate. Previous studies have reported that sodium lauryl sulfate can cause severe dermal irritation and sensitization [47]. The absence of skin sensitization to the treatment of* C. martini* EO topical formulation at 5% may help easy clinical application of the product for the treatment of skin diseases. This supports the traditional claim of using the* C. martini* EO as aromatherapy since the time of antiquity to this day, since its use could have been terminated long time ago if there was skin irritation issue from the use of the oil [48].

All the* C. martini* formulations were optimal in terms of their appearance, homogeneity creaming, and coalescence test and centrifugation test. However, only the microbiologically active formulations, hydrophilic ointment and macrogol blend ointment, had shown good release. Previous studies [24] indicated that both formulations had good release activity against different microorganism and EO.

## 5. Conclusion

Result of current study indicated that EOs of* C. martini *had strong broad-spectrum antimicrobial activity against skin disease causing microorganisms. Moreover, hydrophilic ointment and macrogol blend ointment containing EOs of* C. martini have* superior antimicrobial activity against the majority of tested organism when compared to their respective commercial topical drugs. In addition, the 5% topical formulation of the EO has showed no visible sign of irritation and sensitization. Therefore, EOs of* C. martini *and topical formulations prepared from the oil could be recommended as alternative topical product to synthetic topical drugs for the treatment of skin infection with superiority activity, less toxicity, and low risk of emergence of drug resistance due to the presence of complex mixture of chemical constituents believed to have different mechanism of action and targets. However, further studies should be conducted to explore the shelf life, chronic toxicity, and clinical study to evaluate its safety and efficacy on human subject.

## Figures and Tables

**Table 1:** Composition of formulation bases used to prepare EO topical formulations.

Ingredients	Composition (%)				
	B1	B2	B3	B4	B5
Cetomacrogol emulsifying wax	-	-	9	-	-
Hard paraffin	-	-	-	5	-
Liquid paraffin	-	-	6	-	-
PEG 4000	-	20	-	-	-
PEG 600	-	80	-	-	-
Propylene Glycol	12	-	-	-	-
Sodium lauryl sulfate	1	-	-	-	-
Stearyl alcohol	25	-	-	5	-
Water	37	-	70	-	-
White soft paraffin	25	-	15	85	100
Wool fat	-	-	-	5	-

B1: hydrophilic ointment base, B2: macrogol blend ointment base, B3: macrogol cream base, B4: simple ointment base, and B5: white petrolatum base.

**Table 2:** Minimum inhibitory concentration (MIC) value of *C. martini* EOs against skin disease causing pathogens.

Type of Organism	Organism	MIC value (*μ*g/ml)	
		Clinical Isolate	Standard Strain
Bacteria	*Staphylococcus aureus *	5	5
	*Streptococcus pyogenes*	5	2.5
	*Pseudomonas aeruginosa*	10	5
	*Klebsiella pneumonia*	10	5

Fungi	*Candida albicans*	1.25	*∗*
	*Aspergillus niger*	2.5	2.5
	*Microsporum canis*	0.65	*∗*
	*Trichophyton mentagrophytes*	0.65	0.65
	*Trichophyton verrucosum*	0.65	*∗*
	*Trichophyton rubrum*	0.65	0.65

*∗*: not done.

**Table 3:** Antibacterial activity of *C. martini* EOs against skin disease causing bacteria using agar well diffusion technique.

Plants	Conc %v/v	Inhibition Zone Diameter (mm), including well diameter (10 mm)						
		Clinical Isolates				Standard strains		
		Sa	Sp	Pa	Kp	Sa	Pa	Kp
*C. martini*	4	23.1±2.5^a^	32.0±0.9^a^	16.0±0.6^a^	15.0±0.6^a^	23.1±2.5^a^	17.8±0.8^a^	18.0±0.6^a^
	2	18.5±1.0^b^	28.0±1.9^b^	11.0±0.6^b^	12.0±1.0^b^	18.5±1.0^b^	13.7±1.0^b^	13.0±1.0^b^
	1	14.5±2.0^c^	18.3±1.2^c^	7.0±0.6^c^	9.0±0.9^c^	14.5±2.0^c^	9.3±0.5^c^	10.0±0.6^c^
	0.5	10.5±0.5^d^	12.5±1.0^d^	3.3±1.2^d^	4.0±1.1^d^	10.5±0.5^d^	4.5±1.0^d^	6.0±0.6^d^
	0.25	3.0±0.9^e^	5.3±0.8^e^	0.0±0.0^e^	1.0±0.6^e^	3.0±0.9^e^	0.6±0.5^e^	1.0±0.6^e^
Control	0.2	0.0±0.0^f^	0.0±0.0^f^	0.0±0.0^e^	0.0±0.0^e^	0.0±0.0^f^	0.0±0.0^e^	0.0±0.0^e^

Sa: *S. aureus, *Sp:* S. pyogenes, *Pa: *P. aeruginosa, *Kp: *K. pneumoniae, *Control: 0.2% Tween 80 as negative control, and Conc: concentration. Data (means ± SD, n = 6) within a column with different superscripts are significantly different (p < 0.05).

**Table 4:** Antimycotic activity of *C. martini* EOs against skin disease causing fungal pathogen using agar well diffusion technique.

Plants	Conc %v/v	Inhibition Zone Diameter (mm), including well diameter (10 mm)							
		Clinical Isolates					Standard strains		
		*C.albicans*	*A. niger*	*M. canis*	Tm	*T.verrucosum*	*A. niger*	Tm	*T.rubrum*
*C. martini*	4	31.6±2.5^a^	29.5±0.5^a^	90.0±0.0^a^*∗*	90.0±0.0^a^*∗*	90.0±0.0^a^*∗*	70.2±2.8^a^	90.0±0.0^a^*∗*	90.0±0.0^a^*∗*
	2	30.0±0.6^b^	23.2±0.8^b^	55.0±1.4^b^	90.0±0.0^a^*∗*	65.3±4.0^b^	26.0±2.3^b^	90.0±0.0^a^*∗*	90.0±0.0^a^*∗*
	1	26.0±1.0^c^	17.2±1.2^c^	27.0±0.6^c^	90.0±0.0^a^*∗*	15.5±2.0^c^	19.2±1.5^c^	90.0±0.0^a^*∗*	90.0±0.0^a^*∗*
	0,5	21.2±1.2^d^	12.0±1.8^d^	22.5±1.0^d^	60.2 ±3.0^b^	13.0±0.5^c^	16.3±1.2^c^	60.0±3.6^b^	70.2±3.7^b^
T80	0.2	0.0±0.0^e^	0.0±0.0^e^	0.0±0.0^e^	0.0 ±0.0^c^	0.0±0.0^d^	0.0±0.0^d^	0.0±0.0^c^	0.0±0.0^c^

Ca*: C. albicans, *An:* A. niger, *Mc: *M. canis*, Tm:* T. mentagrophytes, *Tr:* T. rubrum, *Tv:* T verrucosum, *Conc: concentration, T80: 2% Tween 80 as negative control, and *∗*: inhibition of the growth across the entire 90 mm plate. Data (means ± SD, n = 6) within a column with different superscripts are significantly different (p < 0.05).

**Table 5:** Antibacterial activity of topical formulations containing *C. martini* EOs against skin disease causing bacteria using agar well diffusion technique.

Formulations	Conc %v/v	Inhibition Zone Diameter (mm), including well diameter (10 mm)						
		Clinical Isolates				Standard strains		
		Sa	Sp	Pa	Kp	Sa	Pa	Kp
F1	2	28.0±0.9^a^	33.3±0.5^a^	16.0±0.6^a^	15.5±1.0^a^	19.5±1.0^b^	16.0±0.6^a^	18.3±0.5^a^
	1	21.0±0.0^c^	31.3±0.8^b^	8.8±0.8^c^	12.3±1.2^b^	15.5±2.0^d^	9.1±1.5^b^	16.5±1.0^b^
	0.5	19.2±0.6^d^	11.0±0.6^e^	5.0±0.6^e^	6.0±1.0^d^	11.5±0.5^f^	4.0±1.0^c^	6.5 ±1.0^d^
F2	2	17.1± .8^e^	19.2±0.8^d^	13.0±0.6^b^	14.3±0.8^a^	17.0±0.9^c^	15.0±0.6^a^	11.8±1.2^b^
	1	14.0±0.6^f^	11.0±0.9^e^	5.0±0.8^e^	4.0±1.1^e^	13.0±0.5^e^	7.3±0.5^b^	5.3± 1.0^d^
	0.5	6.0±0.6^g^	3.1±1.1^f^	2.0±0.6^d^	1.0±0.0^f^	7.0±0.6^g^	3.0±0.6^c^	2.0±0.0^e^
F3-5	2	0.0±0.0^h^	0.0±0.0^g^	0.0±0.0^f^	0.0±0.0^g^	0.0±0.0^h^	0.0±0.0^d^	0.0±0.0^g^
	1	0.0±0.0^h^	0.0±0.0^g^	0.0±0.0^f^	0.0±0.0^g^	0.0±0.0^h^	0.0±0.0^d^	0.0±0.0^g^
	0.5	0.0±0.0^h^	0.0±0.0^g^	0.0±0.0^f^	0.0±0.0^g^	0.0±0.0^h^	0.0±0.0^d^	0.0±0.0^g^
B1	-	4.0±0.0^h^	6.0±0.0^g^	0.0±0.0^f^	1.0±0.0^f^	4.0±0.0^h^	0.0±0.0^d^	1.0±0.0^f^
B2-5	-	0.0±0.0^h^	0.0±0.0^g^	0.0±0.0^f^	0.0±0.0^g^	0.0±0.0^h^	0.0±0.0^d^	0.0±0.0^g^
Gent	1%	24.0±0.0^b^	25.0±0.0^c^	5.6±0.0e	9.0±0.0^c^	30.0±0.0^a^	7.6±0.8^b^	9.6±0.8^c^

Sa: *S. aureus, *Sp:* S. pyogenes, *Pa: *P. aeruginosa, *Kp: *K. pneumoniae,*-: base only (no EO), B1: hydrophilic ointment base, B2: macrogol blend ointment base, B3: macrogol cream ointment base, B4: simple ointment base, B5: white petrolatum ointment base, F1: hydrophilic ointment, F2: macrogol blend ointment, F3: macrogol cream ointment, F4: simple ointment, F5: white petrolatum ointment, and Gent: gentamycin cream 1% as positive control. Data (means ± SD, n = 6) within a column with different superscripts are significantly different (p < 0.05).

**Table 6:** Antifungal activity of different topical formulations containing *C. martini* EOs using agar well diffusion technique.

Formulation	Conc %V/V	Inhibition Zone Diameter (mm), including well diameter (10 mm)							
		Clinical Isolates					Standard strains		
		*C.albicans*	*A. niger*	*M. canis*	Tm	*T.verrucosum*	*A. niger*	Tm	*T.rubrum*
F1	2	52.2±2.3^a^	30.0±1.2^a^	90.0±0.0^a^*∗*	140.0±0.0^a#^	90.0±0.0^a^*∗*	40.2±1.5^a^	140.0±0.0^a#^	140.0±0.0^a#^
	1.5	47.0±0.9^b^	29.5±1.4^a^	70.0±1.9^b^	140.0±0.0^a#^	90.0±0.0^a^*∗*	28.7±0.9^c^	140.0±0.0^a#^	140.0±0.0^a#^
	1	45.2±2.5^b^	21.0±0^d^	50.5±1.5^d^	90.0±0.0^b*∗*^	48.0±1.4^c^	23.3±1.2^de^	140.0±0.0^a#^	70.2±0.4^d^
F2	2	39.0±1.7^c^	23.0±1.4^c^	90.0±0.0^a^*∗*	140.0±0.0^a#^	90.0±0.0^a^*∗*	37.0±1.4^b^	140.0±0.0^a#^	98.3±11.7^b^
	1.5	37.0±1.2^c^	21.0±1.3^d^	64.1.0±1.4^c^	140.0±0.0^a#^	82.5±1.9^b^	25.0±0.9^d^	140.0±0.0^a#^	85.0±6.2^c^
	1	33.0±0.9^d^	19.0±0^e^	45.0±4.0^e^	42.0 ±1.4^d^	43.0±2.4^d^	22.0±1.8^e^	140.0±0.0^a#^	38.5±2.8^e^
F3-5	2	0.0±0.0^g^	0.0±0.0^g^	0.0±0.0^h^	0.0±0.0^f^	0.0±0.0^f^	0.0±0.0^g^	0.0±0.0^d^	0.0±0.0^g^
	1.5	0.0±0.0^g^	0.0±0.0^g^	0.0±0.0^h^	0.0±0.0^f^	0.0±0.0^f^	0.0±0.0^g^	0.0±0.0^d^	0.0±0.0^g^
	1	0.0±0.0^g^	0.0±0.0^g^	0.0±0.0^h^	0.0±0.0^f^	0.0±0.0^f^	0.0±0.0^g^	0.0±0.0^d^	0.0±0.0^g^
B1	-	4.0±0.0^f^	5.0±0.0^f^	7.0±0.0^g^	11.0±0.0^e^	10.0±0.0^e^	6.0±0.0^f^	13.0±0.0^c^	13.0±0.0^f^
B2-5	-	0.0±0.0^g^	0.0±0.0^g^	0.0±0.0^h^	0.0±0.0^f^	0.0±0.0^f^	0.0±0.0^g^	0.0±0.0^d^	0.0±0.0^g^
Micon	2%	30±0.0^e^	25±1.0^b^	39.0±1.0^f^	45.0±0.0^c^	41±0.0^d^	25±0.0^d^	46.3±1.0^b^	44.0 ±1.0^e^

Tm:* T. mentagrophytes, *Conc: concentration, -: base only (no EO), *∗*: inhibition of the growth across the entire 90 mm plate, ^#^: inhibition of the growth across the entire 140 mm plate, B1: hydrophilic ointment base, B2: macrogol blend ointment base, B3: macrogol cream ointment base, B4: simple ointment base, B5: white petrolatum ointment base, F1: hydrophilic ointment, F2: macrogol blend ointment, F3: macrogol cream ointment, F4: simple ointment, and F5: white petrolatum ointment. Data (means ± SD, n = 6) within a column with different superscripts are significantly different (p < 0.05).

**Table 7:** Antifungal effect of *C. martini *EO at different temperature against selected skin disease causing fungi at their respective MIC using poisoned food technique.

Type of extract	Temp	Inhibition Zone (mm), including well diameter (10 mm)							
		Clinical Isolates					Standard strains		
		*C. albicans*	*A. niger*	*M. canis*	Tm	Tv	*A. niger*	Tm	*T. rubrum*
*C. martini*	100°C	100.0±0.0	100.0±0.0	100.0±0.0	100.0±0.0	100.0±0.0	100.0±0.0	100.0±0.0	100.0±0.0
	80°C	100.0±0.0	100.0±0.0	100.0±0.0	100.0±0.0	100.0±0.0	100.0±0.0	100.0±0.0	100.0±0.0
	60°C	100.0±0.0	100.0±0.0	100.0±0.0	100.0±0.0	100.0±0.0	100.0±0.0	100.0±0.0	100.0±0.0
	40°C	100.0±0.0	100.0±0.0	100.0±0.0	100.0±0.0	100.0±0.0	100.0±0.0	100.0±0.0	100.0±0.0
	RT	100.0±0.0	100.0±0.0	100.0±0.0	100.0±0.0	100.0±0.0	100.0±0.0	100.0±0.0	100.0±0.0

RT: room temperature, Temp: temperature, Tm:* T. mentagrophytes,* andTv:* T. verrucosum *

**Table 8:** Grading scale for the evaluation of challenge patch test reactions in the Guinea Pig Maximization Test of *C. martini *EO topical formulations.

Formulations	Sex	No. of animals	% of test substance	Amount applied	MKGS Result			
					0	1	2	3
Hydrophilic Ointment	F	5	5%	0.5gm	√	-	-	-
	M	5	5%	0.5gm	√	-	-	-
Macrogol Blend Ointment	F	5	5%	0.5gm	√	-	-	-
	M	5	5%	0.5gm	√	-	-	-
Positive Control Group (SLS)	F	5	10%	0.5gm	-	-	-	3/5
	M	5	10%	0.5gm	-	-	-	4/5
Hydrophilic Ointment Base	F	5	Base only	0.5gm	-	1/5	-	-
	M	5	Base only	0.5gm	√	-	-	-
Macrogol Blend Ointment Base	F	5	Base only	0.5gm	√	-	-	-
	M	5	Base only	0.5gm	√	-	-	-

0: no visible change, 1: discrete or patchy erythema, 2: moderate and confluent erythema, 3: intense erythema and swelling, MKGS: Magnusson and Kligman Grading Scale, and SLS: sodium lauryl sulfate

**Table 9:** Physicochemical evaluation of *C. martini *EO topical formulations (n=3).

Property	B1	B2	B3	B4	B5
Homogeneity	Homogenous	Homogenous	Homogenous	Homogenous	Homogenous
Creaming and coalescence	Stable	Stable	Stable	Stable	Stable
Centrifugation test	Stable	Stable	Stable	Stable	Stable
Thermal cycle	Stable	Stable	Separated	Separated	Stable
Freezing and thawing	Stable	Stable	Separated	Separated	Stable
Ph	5.8± 0.4	6.21± 0.01	*∗*	*∗*	*∗*
Fluidity	viscous	M viscous	viscous	M viscous	M viscous
Appearance	soft and fatty	fatty	Very soft and fatty	Clear, good flow	Clear, good flow
Texture	Smooth	Smooth	Smooth	Smooth	Smooth
Color	White	White	White	Opaque	Opaque
Spreadability (g.cm/sec)	14.7	10.5	*∗*	*∗*	*∗*
Weight loss	Nil	Nil	Nil	Nil	Nil
Release	Good	Good	Poor	Poor	Poor

*∗*: not determined, B1: hydrophilic ointment base, B2: macrogol blend ointment base, B3: macrogol cream ointment base, B4: simple ointment base, and B5: white petrolatum ointment base.

## Data Availability

The data used to support the findings of this study are available from the corresponding author upon request.

## References

[1] Monteiro-Riviere N. A.-R., Nancy A. (2010). Structure and function of skin. *Toxicology of the Skin*.

[2] Abubakar E.-M. M. (2009). The use of *Psidium guajava* Linn. in treating wound, skin and soft tissue infections. *Scientific Research and Essays*.

[3] Esposito S., Noviello S., Leone S. (2016). Epidemiology and microbiology of skin and soft tissue infections. *Current Opinion in Infectious Diseases*.

[4] Hollestein L. M., Nijsten T. (2014). An insight into the global burden of skin diseases. *Journal of Investigative Dermatology*.

[5] Afsar F. S. (2010). Skin infections in developing countries. *Current Opinion in Pediatrics*.

[6] Hay R. J., Johns N. E., Williams H. C. (2013). The global burden of skin disease in 2010: an analysis of the prevalence and impact of skin conditions. *Journal of Investigative Dermatology*.

[7] Bongomin F., Gago S., Oladele R., Denning D. (2017). Global and multi-national prevalence of fungal diseases—estimate precision. *Journal of Fungi*.

[8] Basra M. K., Shahrukh M. (2014). Burden of skin diseases. *Expert Review of Pharmacoeconomics & Outcomes Research*.

[9] Marrone R., Vignally P., Rosso A. (2012). Epidemiology of skin disorders in Ethiopian children and adolescents: An analysis of records from the Italian Dermatological Centre, Mekelle, Tigray, Ethiopia, 2005 to 2009. *Pediatric Dermatology*.

[10] Stevens D. L., Bisno A. L., Chambers H. F. (2014). Executive summary: practice guidelines for the diagnosis and management of skin and soft tissue infections: 2014 update by the infectious diseases society of America. *Clinical Infectious Diseases*.

[11] Dryden M. S. (2014). Novel antibiotic treatment for skin and soft tissue infection. *Current Opinion in Infectious Diseases*.

[12] Amirthalingam S., Yi K. S., Ching L. T., Mun N. Y. (2015). Topical antibacterials and global challenges on resistance development. *Tropical Journal of Pharmaceutical Research*.

[13] Carter D., Charlett A., Conti S. (2017). A risk assessment of antibiotic pan-drug-resistance in the UK: bayesian analysis of an expert elicitation study. *Antibiotics*.

[14] WHO The world is running out of antibiotics, WHO report confirms. http://www.who.int/news-room/detail/20-09-2017-the-world-is-running-out-of-antibiotics-who-report-confirms.

[15] Gupta P. D., Birdi T. J. (2017). Development of botanicals to combat antibiotic resistance. *Journal of Ayurveda and Integrative Medicine*.

[16] Savoia D. (2012). Plant-derived antimicrobial compounds: alternatives to antibiotics. *Future Microbiology*.

[17] Kassaye K. D., Amberbir A., Getachew B., Mussema Y. (2006). A historical overview of traditional medicine practices and policy in Ethiopia. *Ethiopian Journal of Health Development*.

[18] Tadele A., Gemeda N., Tadele A., Giram B., Teka F. Development of broad spectrum dermatological from some herbal remedies.

[19] Mohammed A. Y. (2016). Knowledge, attitude and practice of community on traditional medicine in Jara Town, Bale Zone South East Ethiopia. *Science Journal of Public Health*.

[20] Singh V. K., Singh P. K., Sharma P. K., Srivastava P. K., Mishra A. (2013). Formulation and evaluation of topical gel of acelofenac containing piparine. *Indo American Journal of Pharmaceutical Research*.

[21] Das K., Tiwari R., Shrivastava D. (2010). Techniques for evaluation of medicinal plant products as antimicrobial agents: current methods and future trends. *Journal of Medicinal Plants Research*.

[22] Balouiri M., Sadiki M., Ibnsouda S. K. (2016). Methods for *in vitro* evaluating antimicrobial activity: a review. *Journal of Pharmaceutical Analysis*.

[23] Rodeheaver G. T., Gentry S., Saffer L., Edlich R. F. (2008). Topical antimicrobial cream sensitivity testing. *Surgery, Gynecology, and Obstetrics*.

[24] Gemeda N., Urga K., Tadele A., Lemma H., Melaku D., Mudie K. Antimicrobial activity of topical formulation containing eugenia caryophyllata L. (Krunfud) and myritus communis L. (Ades) essential oils on selected skin disease causing microorganisms. *EthiopianJournal of Health Sciences*.

[25] Shahi S. K., Shahi M. P. Broad spectrum herbal therapy against superficial fungal infections.

[26] OECD (1992). Skin Sensitisation. *OECD Guideline for Testing of Chemicals*.

[27] Buehler E. V., Kreuzmann J. J., Sakr A., Gu X. H. (1990). Comparable sensitivity of hairless and hartley strain guinea pigs to a primary irritant and a sensitizer. *Journal of Toxicology - Cutaneous and Ocular Toxicology*.

[28] Food and D. Administration (2005). Guidance for industry: estimating the maximum safe starting dose in initial clinical trials for therapeutics in adult healthy volunteers. *Center for Drug Evaluation and Research (CDER)*.

[29] Hamedi A., Zarshenas M. M., Sohrabpour M., Zargaran A. (2013). Herbal medicinal oils in traditional Persian medicine. *Pharmaceutical Biology*.

[30] Ali B., Al-Wabel N. A., Shams S., Ahamad A., Khan S. A., Anwar F. (2015). Essential oils used in aromatherapy: a systemic review. *Asian Pacific Journal of Tropical Biomedicine*.

[31] Dhifi W., Bellili S., Jazi S., Bahloul N., Mnif W. (2016). Essential oils’ chemical characterization and investigation of some biological activities: a critical review. *Medicines*.

[32] Raina V. K., Srivastava S. K., Aggarwal K. K., Syamasundar K. V., Khanuja S. P. (2003). Essential oil composition of Cymbopogon martini from different places in India. *Flavour and Fragrance Journal*.

[33] Mahdavi B., Yaacob W. A., Din L. B. (2017). Chemical composition, antioxidant, and antibacterial activity of essential oils from Etlingera sayapensis A.D. Poulsen & Ibrahim. *Asian Pacific Journal of Tropical Medicine*.

[34] Fde O. P., Mendes J. M., Lima I. O., Mota K. S., Oliveira W. A., Ede O. L. (2015). Antifungal activity of geraniol and citronellol, two monoterpenes alcohols, against Trichophyton rubrum involves inhibition of ergosterol biosynthesis. *Pharmaceutical Biology*.

[35] Ternus Zr Z. M. (2015). Microbiological characterization of pure geraniol and comparison with bactericidal activity of the cinnamic acid in gram-positive and gram-negative bacteria. *Journal of Microbial & Biochemical Technology*.

[36] Nazzaro F., Fratianni F., Coppola R., Feo V. D. (2017). Essential oils and antifungal activity. *Pharmaceuticals*.

[37] Bansod S., Rai M. (2008). Antifungal activity of essential oils from Indian medicinal plants against human pathogenic Aspergillus fumigatus and A. niger. *World Journal of Medical Sciences*.

[38] Gemeda N., Woldeamanuel Y., Asrat D., Debella A. (2014). Effect of Cymbopogon martini, Foeniculum vulgare, and Trachyspermum ammi essential oils on the growth and mycotoxins production by Aspergillus species. *International Journal of Food Science*.

[39] Lodhia M. H., Bhatt K. R., Thaker V. S. (2009). Antibacterial activity of essential oils from palmarosa, evening primrose, lavender and tuberose. *Indian Journal of Pharmaceutical Sciences*.

[40] Nazzaro F., Fratianni F., De Martino L., Coppola R., De Feo V. (2013). Effect of essential oils on pathogenic bacteria. *Pharmaceuticals*.

[41] Aelenei P., Miron A., Trifan A., Bujor A., Gille E., Aprotosoaie A. (2016). Essential oils and their components as modulators of antibiotic activity against gram-negative bacteria. *Medicines*.

[42] Randriamiharisoa R. P., Gaydou E. M. (1987). Composition of palmarosa (Cymbopogon martini) essential oil from madagascar. *Journal of Agricultural and Food Chemistry*.

[43] Florence A. T., Attwood D. (1998). *Physicochemical Principles of Pharmacy*.

[44] Ng K. (2018). Penetration enhancement of topical formulations. *Pharmaceutics*.

[45] Berghe D. A. V., Vlietinck A. J., Hostettmann K. (1991). Screening methods for antibacterial and antiviral agents from higher plants. *Methods in Plant Biochemistry*.

[46] Mansour A., Enayat K., Neda M.-S., Behzad A. (2010). Antibacterial effect and physicochemical properties of essential oil of Zataria multiflora Boiss. *Asian Pacific Journal of Tropical Medicine*.

[47] Bondi C. A., Marks J. L., Wroblewski L. B., Raatikainen H. S., Lenox S. R., Gebhardt K. E. (2015). Human and environmental toxicity of sodium lauryl sulfate (SLS): evidence for safe use in household cleaning products. *Environmental Health Insights*.

[48] Mercola J. *Palmarosa oil: a potent way to improve skin health*.

